# Biodegradation of COVID19 antibiotic; azithromycin and its impact on soil microbial community in the presence of phenolic waste and with temperature variation

**DOI:** 10.1007/s11274-023-03591-7

**Published:** 2023-04-11

**Authors:** Shaimaa Abd El Mohsen Ibrahim, Heba Abdalla El-Bialy, Ola M. Gomaa

**Affiliations:** Radiation Microbiology Department, National Center for Radiation Research and Technology (NCRRT), Egyptian Atomic Energy Authority (EAEA), 3 Ahmad El Zomor St, Cairo, Egypt

**Keywords:** Antibiotic, Soil, Microbial community, Phenolic waste, Biodegradation, COVID19

## Abstract

The increase in using antibiotics, especially Azithromycin have increased steadily since the beginning of COVID19 pandemic. This increase has led to its presence in water systems which consequently led to its presence upon using this water for irrigation. The aim of the present work is to study the impact of irrigation using Azithromycin containing water on soil microbial community and its catabolic activity in the presence of phenolic wastes as compost. Wild berry, red grapes, pomegranate, and spent tea waste were added to soil and the degradation was monitored after 5 and 7 days at ambient and high temperatures. The results obtained show that at 30 °C, soil microbial community collectively was able to degrade Azithromycin, while at 40 °C, addition of spent tea as compost was needed to reach higher degradation. To ensure that the degradation was biotic and depended on degradation by indigenous microflora, a 25 kGy irradiation dose was used to kill the microorganisms in the soil and this was used as negative control. The residual antibiotic was assayed using UV spectroscopy and High Performance Liquid Chromatography (HPLC). Indication of Azithromycin presence was studied using Fourier Transform Infrared Spectroscopy (FTIR) peaks and the same pattern was obtained using the 3 used detection methods, the ability to assign the peaks even in the presence of soil and not to have any overlaps, gives the chance to study this result in depth to prepare IR based sensor for quick sensing of antibiotic in environmental samples.

**Supplementary Information:**

The online version contains supplementary material available at 10.1007/s11274-023-03591-7.

## Introduction

The consumption of antibiotics has increased over the past 30 years. About 50 to 90% of the antibiotics consumed are excreted as a mixture of parent compounds and bioactive metabolites, eventually, they reach open water bodies. The exposure to small amounts of antibiotics over the long-time results in compromising of human health through disruption of the endocrine system and production of Antibiotic Resistant bacteria (Wang et al 2021). After the extensive use of antibiotics during COVID 19 pandemic, it was expected that azithromycin will be present in irrigation water. Azithromycin belongs to the macrolides antibiotic class which depends on inhibition of protein synthesis by reversibly binding to 50S ribosomal RNA subunit as a mode of action. It is commonly used by humans for respiratory tract infection (Grenni et al 2018, Jafari Ozumchelouei et al. 2020). A study performed recently reported that the presence of azithromycin in Persian Gulf area has increased in treatment plants after COVID19 and reached up to 48 times (Mirzaie et al 2022). Antibiotics reside eventually in agriculture, aquaculture and treatment plants (Saravanan et al 2022). The fate and removal of antibiotic in soil is determined by the extend of adsorption/desorption and biodegradation by indigenous microbial community (Conde-Cid et al 2020). The increase in antibiotics is expected to affect soil microbial community and in return will affect microbial performance and catabolic activity, especially in the presence of other contaminants (Liang et al 2022). There is a potential risk in the release of antibiotics on soil, consequently entering the food chain which will affect agriculture and eventually human health (Conde-Cid et al 2020).

The study of microbiome sheds light on the indigenous microbial community present in a certain environment and in the presence of a certain pollutant. This reflects on how we can manage the bioremediation process. Information generated from studying the microbiome and produced metabolites can add to the tailoring of the bioremediation process to maximize the process while cutting down the cost. Recent research studies have shown that the use of bacterial consortia proved better efficiency in the degradation of dyes than individual bacterial isolates (Krithika et al. 2021). In addition, the use of the consortium achieves a one-pot treatment regimen, adding to this pot compounds that can assist the consortium will enhance the bacterial catabolic activity and ensure balance to the ecosystem. This can be attributed to the presence of several synergistic metabolic networks created by consortia compared to pure individual microbial isolates.

Recent changes in climate have led to changes in microbial activity in ecosystem, it is expected that the combined effect of temperature and antibiotic presence in soil would have a profound effect and will induce changes in the catabolic performance and microbial community in soil. From this standpoint, the aim of the present work is to study the impact of Azithromycin containing water on soil indigenous bacterial community and its catabolic activity in the presence of phenolic wastes at 30 and 40 °C.

## Materials and methods

### Soil samples and incubation conditions

A soil sample was taken from one of the gardens located at National Center for Radiation Research and Technology (NCRRT) premises in Nasr City, Cairo, Egypt. The garden was cultivated with ornamental flowers and date palms. The soil sample was collected at the depth of 15–20 cm (near the rhizosphere area of 12 years old palm tree), placed in sterile polyethylene bags, and stored at 4 °C until use. The soil sample was divided into portions; 5 g each. A soil samples was irradiated with 25 kGy using Indian Gamma Chamber 4000 A at a dose rate; 0.725 kGy/h and served as a control (sample with no viable bacteria). A final concentration of 10,000 ppm/kg soil azithromycin (Azithromycin®, Pfizer, USA) was added to soil by irrigating the soil portions. Individual portions (with the antibiotic) were incubated at 30 °C and 40 °C in the presence of phenolic wastes and samples were taken after 0 and 7 days to assay the total bacterial count. The results are reported as LogN.

### The phenolic wastes

About 0.1 g phenolic waste compound/1 g soil of wild berry, pomegranate, and red-grape fruits were purchased from local markets in Cairo, and fruits’ pomaces were recovered after squeezing out juices. In addition, spent tea waste was collected from local café shops and restaurants in Cairo and Giza governorates. Afterward, the wastes were dehydrated at 60 °C for 6 h and stored at 4 °C until use (El-Bialy and Abd El-Aziz 2009).

### Quantification of azithromycin biproducts

At the end of the incubation time, the antibiotic under investigation was extracted and quantified and the microbial load of soil samples was determined using standard protocols (Wolf 2006). In a preliminary experiment, the microbial load of soil samples received azithromycin at 1000 or 10,000 ppm/kg soil was determined after 5 and 7 days to determine the optimum time using three approaches:

#### UV–visible spectroscopy

The azithromycin antibiotic was extracted from the soil samples using acetonitrile as previously described (Miranda et al. 2019) and determined by acidic hydrolysis with 27N HCl and reading the absorbance at 482 nm using a T60 UV–Vis spectrophotometer (Haleem et al. 2006). The absorbance readings were converted to azithromycin concentrations regarding a standard curve that was done using increasing concentrations of azithromycin (0.1–0.5 µg/mL).

#### HPLC analysis

The treatments and replicates were harvested after 7 days. Azithromycin was extracted by adding different amounts of antibiotic dissolved in DMSO and completing to 1000 µl with HPLC grade methanol. The extracts were filtered directly into amber (0.22 m$\mu$) HPLC vilas. The conditions for the antibiotic detection and quantification were as follows:

Mobile phase: Methanol (50%): Acetonitrile (50%), Column: 120CC-C18 column (Poroshell 120, length 100 mL, diameter 4.6 mm, particle size 2.7 micron; 600 Bar), Temperature of column 25 ºC Flow rate: 1 mL min^−1^, Retention time: Approximately 1.179, Wavelength 240 nm. 100 µl of the extracted sample was suspended in a mobile phase, filtered through a 0.45 µm membrane filter. The column was equilibrated for at least 1 h with mobile phase flowing through the chromatographic system before starting the assay. About 5 µl of the standard or sample solution were injected into the chromatograph using conditions described above. Considering the possibility of using this analytical procedure in stability studies, concentrations 15, 30, 60, 125, 250 mg/mL were prepared by dissolving azithromycin in mobile phase, in order to study system linearity response. The regression curve of peak areas versus concentrations proved linear with a coefficient of correlation r = 0.9993 and with confidence intervals at *P* = 0.05, Y = 17.363x.

#### Fourier transform infrared spectroscopy (FT-IR)

Fourier Transform Infrared Spectroscopy (FT-IR) of soil samples containing Azithromycin were added directly for ATR-FTIR analysis. Scanning was performed from 400 to 4000 nm using ATR-FTIR, BRUKER VERTEX 70 optics layout device at NCRRT. The analytical spectrum was then compared to the library to identify the functional groups.

### Microbiome analysis

The microbial DNA was extracted using Qiagen DNeasy powerMax Soil kit® according to manufacturer’s instructions. The 16S metagenomics library preparation kit includes 2 sets of primers that correspond to the hypervariable regions of the 16S rDNA gene in bacteria. The primer sets were V2-4–8, V3-6 and V7-9. The sequencing was carried out at Colours Medical laboratory (Maadi, Egypt) using IonTorrent™ Next Generation Sequencer. Diversity metrics were calculated using core-metrics-phylogenetics. QIIME2 was used to visualize the results in addition to R packages phyloseq and ggplot2. Details of all kits used and links to products are in Supplementary material S1.

## Results

### Growth of indigenous bacterial soil samples in the presence of azithromycin and at two different temperatures

**Fig. 1:**
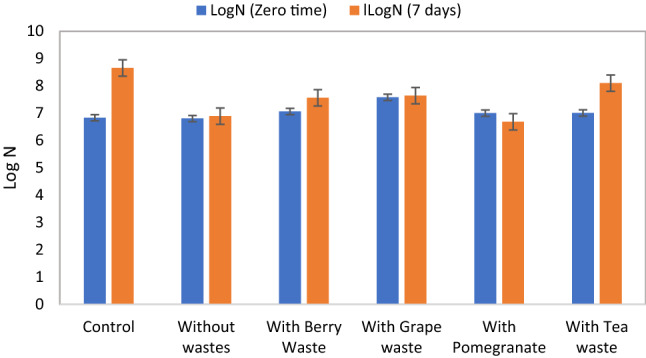
Total soil microbial growth with and without adding phenolic wastes at zero and 7 days of incubation at 30 °C. Control represents a sample without azithromycin

**Fig. 2:**
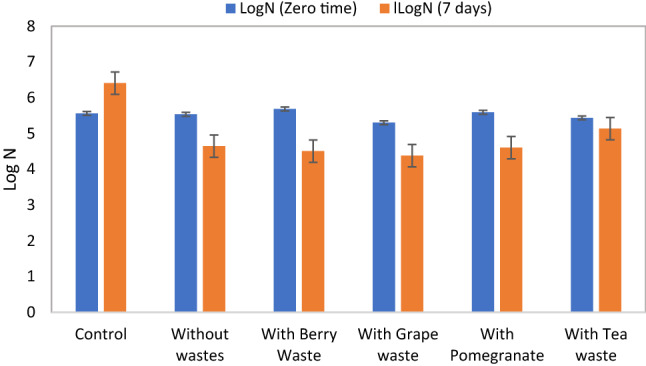
Total soil microbial growth with and without adding phenolic wastes at zero and 7 days of incubation at 40 °C. Control represents a sample without azithromycin

Changes in LogN at zero- and 7-days incubation period were monitored for soil samples grown incubated at 30 and 40 °C. The obtained results show that in the presence of phenolic wastes, the growth has changed, and that the highest growth was shown for soil samples incubated with spent tea waste for samples incubated at 30 °C which increased 1.16-fold after 7 days as compared to 1.26-fold increase for control soil sample without phenolic wastes. The remaining used phenolic wastes showed minimal or low change in LogN after 7 days of incubation (Fig. 1). On the other hand, soil samples incubated at 40 °C showed 1.15-fold increase for control samples, and a 1.05-fold decrease in LogN for soil samples incubated with spent tea waste for samples after 7 days incubation. The remaining phenolic wastes showed more decrease that reached 1.26-fold when added to soil and incubated at 40 °C for 7 days (Fig. 2).

### Degradation of azithromycin at two different temperatures, with phenolic wastes and using different assays

**Fig. 3:**
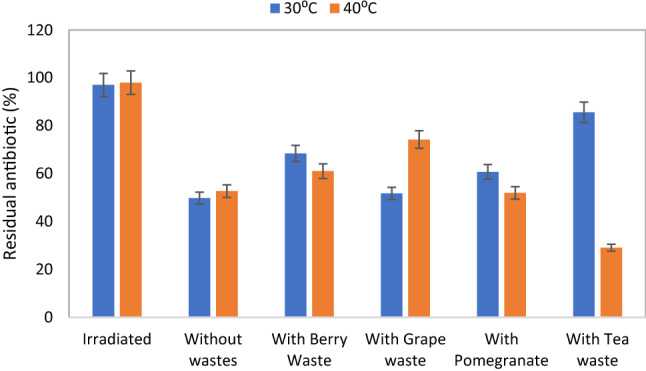
Represent the residual antibiotic in % in soil after incubation with and without phenolic wastes and incubation for 5 days. Gamma irradiation was used as the negative control sample

**Fig. 4:**
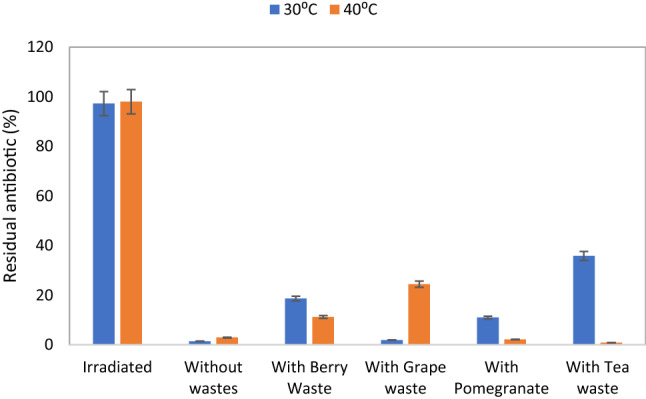
Represent the residual antibiotic in % in soil after incubation with and without phenolic wastes and incubation for 7 days. Gamma irradiation was used as the negative control sample

Examining the degradation pattern of azithromycin was performed in the presence of different phenolic wastes added as phenolic wastes to soil, and at two temperatures. The residual antibiotic was assayed after incubation for 5 and 7 days at 30 and 40 °C, and the data are represented in Fig. 3, 4, respectively. The results show that incubation with different wastes led to discrepancy in the degradation activity of the microbial consortia. The highest degradation can be detected for soil samples with no phenolic waste amendments at 30 °C, while that at 40 °C required the addition of spent tea waste. The pattern of degradation also was observed to be slow for some samples at incubation of 5 days, on the other hand, samples with and without addition of phenolic wastes showed close degradation pattern which indicates that time is a key factor. Highest degradation can be observed for samples incubated with spent tea waste after 5 days at 40 °C which was higher than that observed for samples without phenolic waste amendment or wild berry waste, grape waste, or pomegranate wastes. On the other hand, incubation for 7 days resulted in close results in the degradation percentage for samples incubated with spent tea waste (99.164%) and for samples without any amendments (98.56%). Degradation was confirmed as biotic process since the exposure of soil samples to gamma radiation using the sterilization dose 25 kGy led to no degradation and the degradation was shown to be 2.95 and 2% for samples incubated for 5 days at 30 and 40 °C. While for samples incubated for 7 days the degradation was 2.77 and 2% for incubation at the abovementioned temperatures.

**Fig. 5:**
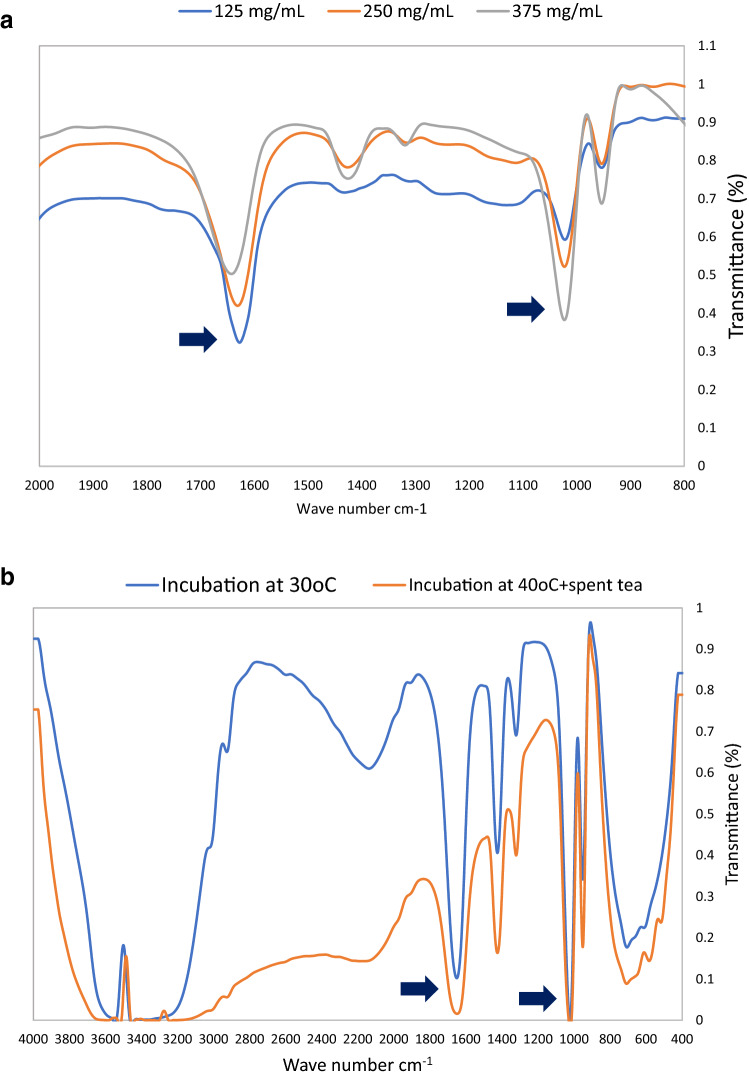
**a**. FTIR spectrum for different concentrations of azithromycin. **b**. Residual azithromycin in soil samples incubated at 30 °C and 40 °C with added spent tea. Analysis is performed using FTIR spectroscopy

FTIR spectrum of azithromycin concentrations of 125, 250 and 375 mg/mL showed a peak at 1625.9 cm-1 and 1010 cm-1 were detected for azithromycin that increased with the increase in azithromycin concentration (Fig. 5a). Extraction of residual azithromycin from soil samples incubated at 30 °C and 40 °C with spent tea showed for samples containing antibiotics showed a decrease in azithromycin characteristic peak for 40 °C with spent tea than control samples incubated at 30 °C (Fig. 5b). The results resonate with the residual azithromycin results shown above in Fig. 4.

**Fig. 6:**
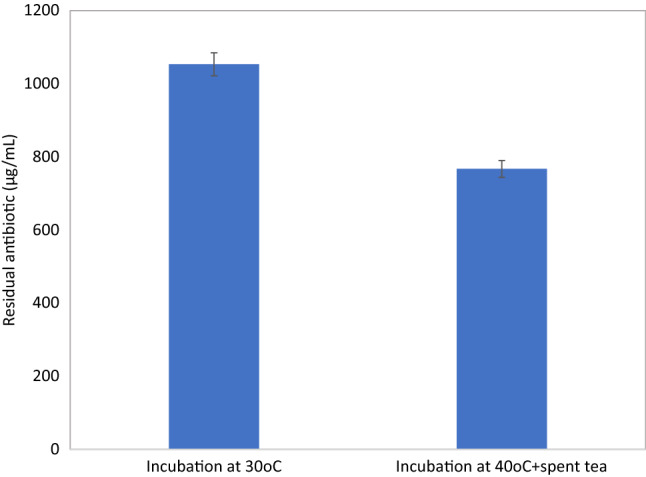
Residual azithromycin in soil samples incubated at 30 °C and 40 °C with added spent tea. Analysis is performed using HPLC and calculated against standard azithromycin sample

Residual azithromycin assayed using HPLC also showed the same pattern as those obtained by UV assay and FTIR, where the residual azithromycin was 1053 µg/mL for soil sample incubated at 30 °C and 766.96 µg/mL for that incubated at 40 °C with spent tea for 7 days (Fig. 6).

### Soil microbial consortium in the samples containing azithromycin at two different temperatures

**Fig. 7:**
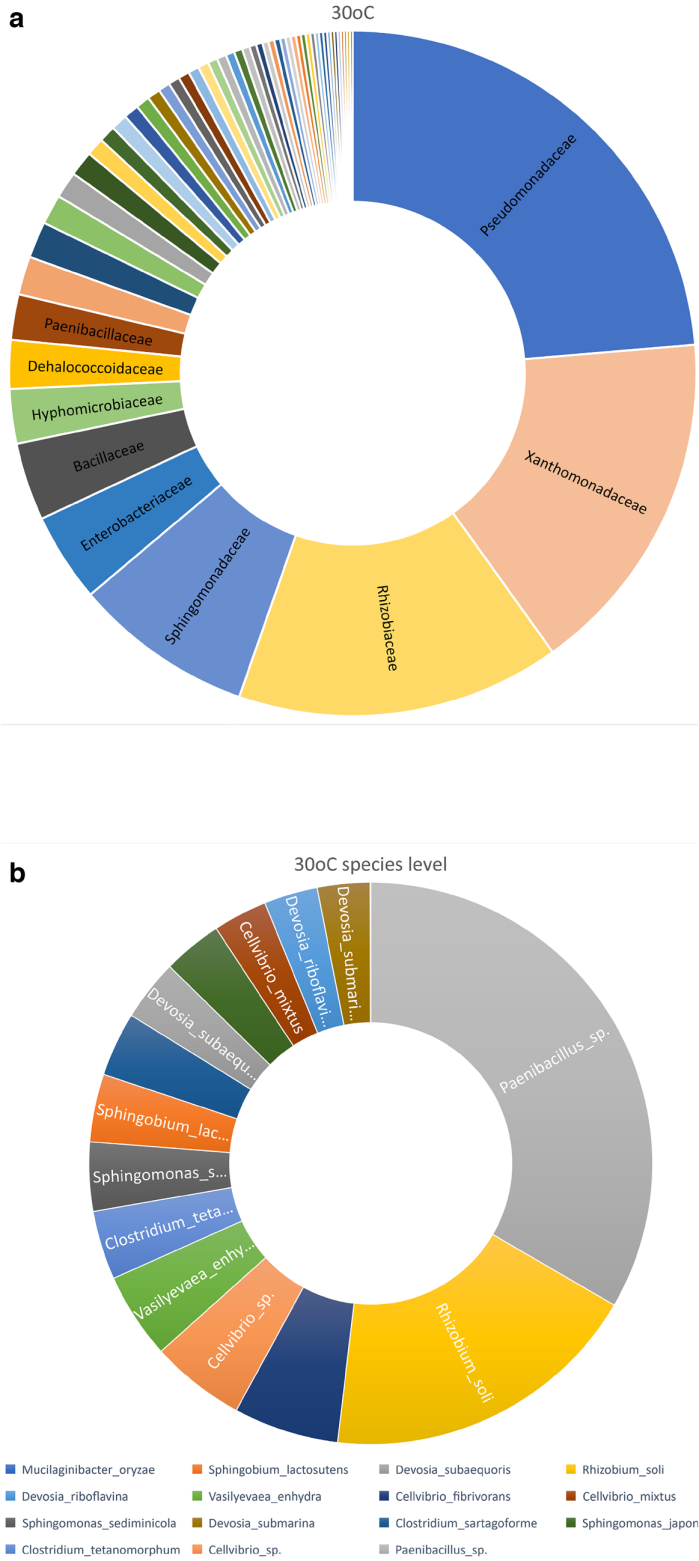
**a**. Relative abundance on family level for soil sample containing azithromycin incubated at 30 °C. The cut off was > 1000. **b**. Relative abundance on species level for soil sample containing azithromycin incubated at 30 °C. The cut off was > 1000

**Fig. 8:**
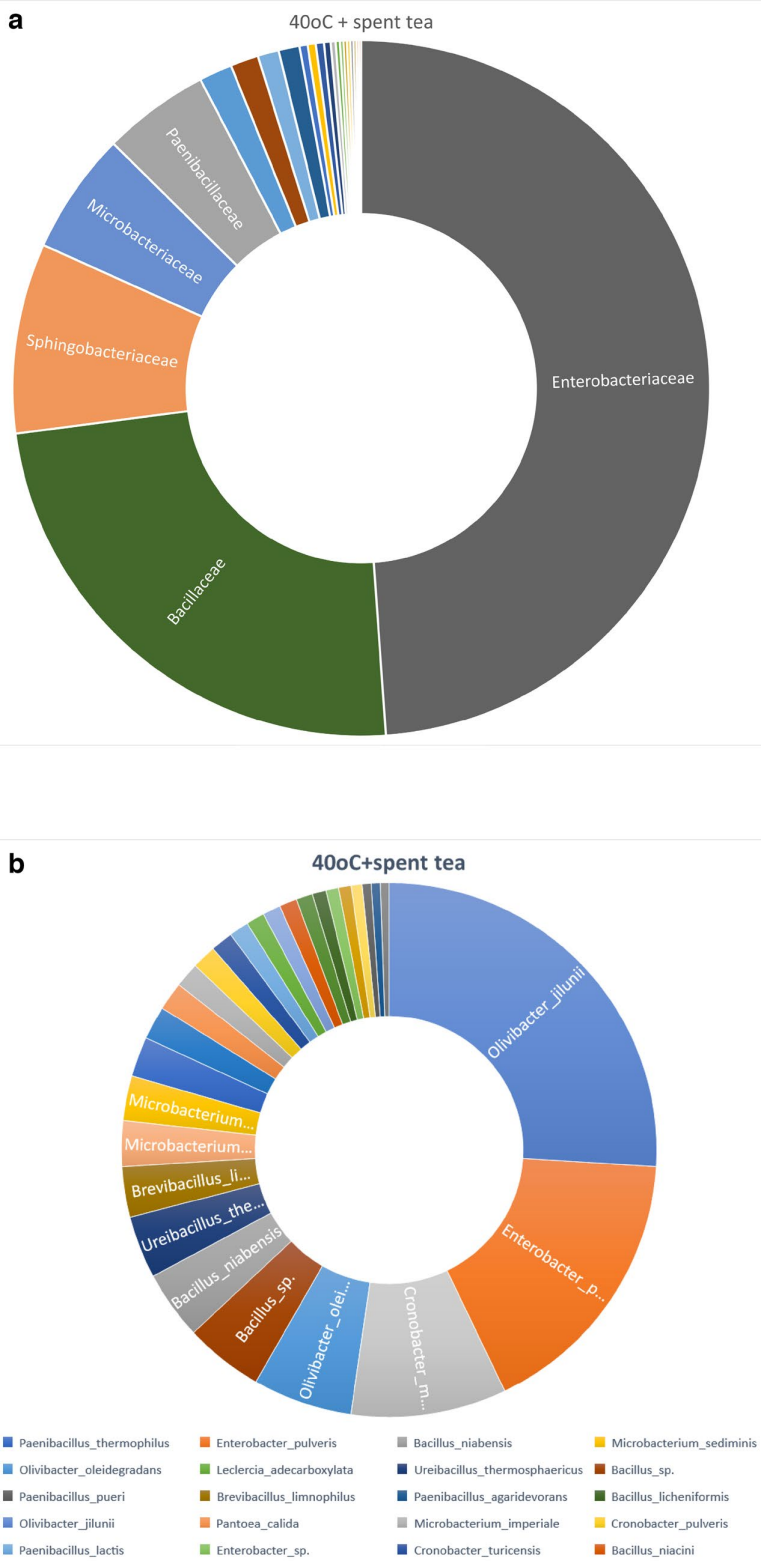
**a**. Relative abundance on family level for soil sample containing azithromycin and spent tea and incubated at 40 °C. The cut off was > 1000. **b**. Relative abundance on species level for soil sample containing azithromycin and spent tea and incubated at 40 °C. The cut off was > 1000

To understand the relationship between the presence of azithromycin and soil microbial community at 30 °C and 40 °C with spent tea, the whole bacterial community was identified at the family and genus level. Dominant families obtained after incubation of soil sample at 30 °C were: Pseudomonadaceae, Rhizobiacaea, Desulfobacteriacea, Deinococcaceae, Bacillaeace, Sphingiomonadaceae. Soil samples incubated at 30 °C Genus majority in descending order are Bacillus, Krasilinkovia, Lysinibacillus, Rhodococcus, Sphingobium, Rubrivivax, Paenibacillus. The relative abundance for families and genus with cut off ˃1000 are represented in Fig. 7a, b. On the other hand, soil samples incubated at 40 °C with spent tea showed dominant families of Enterobacteriaceae, Bacilleacea, Paneibacilleacea, Sphingiobacteriaceae, Bradyrhizobaceae. Genus majority in descending order are Bacillus, Nitrate reducers, Bervibacillus, Microbacterium, Serratia, Paeniebacillus, and Enterobacter. The relative abundance for families and genus with cut off ˃1000 are represented in Fig. 8a, b. The images of complete families and genus for both samples are represented in S2.

## Discussion

The presence of antibiotics in the environment can modify native microbial communities and diversity thereby altering natural biogeochemical cycling, causing potentially detrimental effects on agriculture as well as contributing to the growing worldwide antibiotic resistance epidemic (Maier and Tjeerdema 2018). The present work demonstrates the changes that took place due to the presence of Azithromycin in soil under 2 different temperatures and in the presence of different composts. The results show that the total bacterial community has changes in terms of count and dynamics. The persistence of macrolide antibiotics in the soil depends on the proliferation of biodegrading microorganisms in the soil and is independent of prior exposure to the drug (Topp et al. 2016). The higher initial azithromycin concentration was a key variable for biodegradation kinetics. Although many reports previously described the positive impact of manure addition on the biodegradation of chemicals in agricultural soils, Topp et al. (2016) found the sorptive interactions of azithromycin with the organic matter will reduce macrolide bioavailability for biodegradation. Microbial respiration in azithromycin contaminated soil was significantly greater in the biosolids alone than in the amended manured sand treatments, reflecting the greater organic matter and nutrient contents of biosolids than of the manured sand. Amendment with biosolids (1% w/w) increased the organic matter and nutrient content of the manured sand; but did not significantly affect microbial respiration (Sidhu et al. 2019).

Optimizing time in the removal reactions will save the cost of utilization and energy consumption (Bazrafshan et al. 2013). Terzic et al. (2018) revealed that the elimination efficiency of macrolide antibiotic; azithromycin in activated sludge reached 99% after a prolonged incubation period exceeding 160 h. The half-life of azithromycin in outdoor mesocosms over a period of three years was calculated to be in the range of 770 ± 181 to 11.77 ± 7.34 days when soil-biosolid mixtures were incubated together after previous soil exposures to macrolide antibiotics (Maier and Tjeerdema 2018), although there was no evidence for the accelerated degradation of many pharmaceuticals in Mexican soils that have received untreated wastewater for up to 100 years (Topp et al. 2016).

The elevation of temperature significantly increases the cavitation intensity and leads to an increase in azithromycin ionization and its reduction whereas the concentration of radical hydroxyl deceases at a lower temperature and subsequently decreases the degradation of biocides and pharmaceuticals (Tao, et al., 2015). Yazdani and Sayadi (2018) demonstrated the removal rate of organic compounds is directly proportional to the temperature because organic molecules migrate from the solution to the region where the hydroxyl radical concentration is high.

The typical macrolide antibiotics are relatively large molecules, which consist of a macrocyclic lactone ring containing 14 to 16 atoms, substituted with hydroxyl, alkyl, and ketone groups and with neutral or amino sugars bound to the ring by substitution of hydroxyl groups (Terzic et al. 2018). One of the key initial steps in azithromycin transformation is enzymatic hydrolytic opening of the macrolactone ring, most probably mediated by the enzyme macrolide esterase; this could be the reason for the clinically relevant resistance (Morar et al. 2012). Esterification was formed either by the removal of one or both sugar units and some modification of desosamine sugar moiety (Voigt and Jaeger 2017). This was followed by the formation of the corresponding phosphorylated or glycosylated transformation products (Terzic et al. 2011). Since phosphorylation is a well-known microbial strategy for the inactivation of macrolide antibiotics (Dinos 2017). The macrolactone ring opening was followed by two biotransformation steps including two subsequent water losses, which could have occurred at two different positions. After that, azithromycin mineralization to carbon dioxide and inorganic salts was efficiently biotransformed both under aerobic and anaerobic conditions (Terzic et al. 2018).

The detected FTIR spectra in our work showed peaks characteristic for C–H for methyl groups, C=O group of lactone and C–O. Robaina et al (2013) reported that FTIR can be used to detect azithromycin and that the characteristic is the peak for C=O group of lactone and that it can also be used for quantitative analysis. Miranda et al (2019) also reported the applicability of using spectroscopy coupled with Fourier Transform for the detection of azithromycin. While both used acetonitrile detection of the antibiotic from soil prior to spectroscopical analysis, our work represents the detection directly in soil samples which makes it easier and practical to use if FTIR is coupled to a hand held device. Assi et al (2021) validated the use of portable near infra-red spectroscopy for detection of several groups of antibiotics in their pure form. The results obtained showed an increase in transmittance peaks that were proportional to the azithromycin concentrations in the soil sample. The results also followed the same patterns detected using UV–Visible and HPLC in the present study. This result encourages the use of FTIR directly to soil samples without extraction.

The presence of azithromycin and phenolic wastes in soil incubated at elevated temperature resulted in change in the bacterial community. Although little information has been published with the three tested parameters, the administration of any of these parameters has been known to change the microbiota. Liang et al (2022) reported that the presence of antibiotics changed the kinetics of degradation and dominance of antibiotic resistant bacteria in a biofilm reactor. Cerqueira et al (2020) reported changes in the microbiome and resistome of soil irrigated with three different antibiotics and the prevalence of Xantomonadales species in the root microbiome of lettuce grown in this soil. The presence of fertilizer was reported to manipulate the soil microbiome altering soil resistome (Li et al 2022). In the present study, soil samples incubated at 40 °C with spent tea waste has led to the highest degradation of azithromycin, however, Enterobacteriaceae became predominant which represented almost half the microbial community. The Enterobacteriaceae family is known to include several gram-negative pathogenic bacteria. On the other hand, the microbial community of soil sample incubated at 30 °C without adding the compost resulted in less azithromycin degradation but had several predominant families such as Pseudomonadaceae, Xanthomonadaceae, Rhizobiaceae and Sphingobacteriaceae. This confirms the reports above that changes in soil microbial community can result from additions of antibiotics or other compounds.

## Conclusion

In conclusion, this study highlights that the accumulation of antibiotics in soil leads to disturbance of natural catabolic activity of indigenous microbial community. The dominance of a family over another controls the soil indigenous microbial activity in terms of degradation which can also affect the soil community in terms of plant activity and can be expected to affect the plant growth and/or pathogenesis. The presence of compost also plays a role in soil catabolic activity and more studies are recommended to understand its contribution, especially with climate change. The fact that we can detect the residual antibiotic in soil using FTIR paves the way to preparing an IR sensor for simple detection as opposed to laborious analytical methods and will also reduce time needed for detection. The novel IR sensors are nowadays small and can be coupled with mobile phones. This technology will be the future detection tools of environmental pollutants. More work is in the pipeline to build upon the obtained results.

## Supplementary Information

Below is the link to the electronic supplementary material.

Supplementary file1 (DOCX 425 KB)
