# Supplementary material for: Biodegradation of COVID19 antibiotic; azithromycin and its impact on soil microbial community in the presence of phenolic waste and with temperature variation
Source: World J Microbiol Biotechnol. 2023 Apr 11;39(6):154. doi: 10.1007/s11274-023-03591-7 (PMC10085964; doi:10.1007/s11274-023-03591-7)
Supplement: Supplementary file 1 — Supplementary file1 (DOCX 425 KB) [file 11274_2023_3591_MOESM1_ESM.docx]

S1:

Names of kits

| **No.** | **Step** | **Kit** | **Manufacture** | **Instrument** |
| --- | --- | --- | --- | --- |
| 1 | Amplify the 16S hypervariable regions | Ion 16S™ Metagenomics Kit | ThermoFisher Scientific | 9700 Thermal Cycler |
| 2 | Purify the amplification products | Agencourt™ AMPure™ XP Reagent | Beckman Coulter | DynaMag™-2 Magnet |
| 3 | Calculate DNA input | Qubit dsDNA HS assay | ThermoFisher Scientific | Qubit 3.0 |
| 4 | Ligate barcoded adapters and nick‑repair | Ion Xpress™ Plus Fragment Library Kit /  Ion Xpress™ Barcode Adapters | ThermoFisher Scientific | 9700 Thermal Cycler |
| 5 | Purify the unamplified library | Agencourt™ AMPure™ XP Reagent | Beckman Coulter | DynaMag™-2 Magnet |
| 6 | Quantify the library | Ion Library Taqman Quantitation Kit | ThermoFisher Scientific | ViiA 7 Real Time PCR |
| 7 | Template preparation, loading chips and sequencing | Ion PI™ Hi-Q™ Chef Kit | Ion Torrent | Ion Chef |
| 8 |  | Ion PI™ Chip |  | Ion Proton |

Protocol as mentioned at <https://www.thermofisher.com/order/catalog/product/A26216?SID=srch-srp-A26216>

Sequencing was performed using the following products:

<https://www.thermofisher.com/order/catalog/product/4484177>

S2:


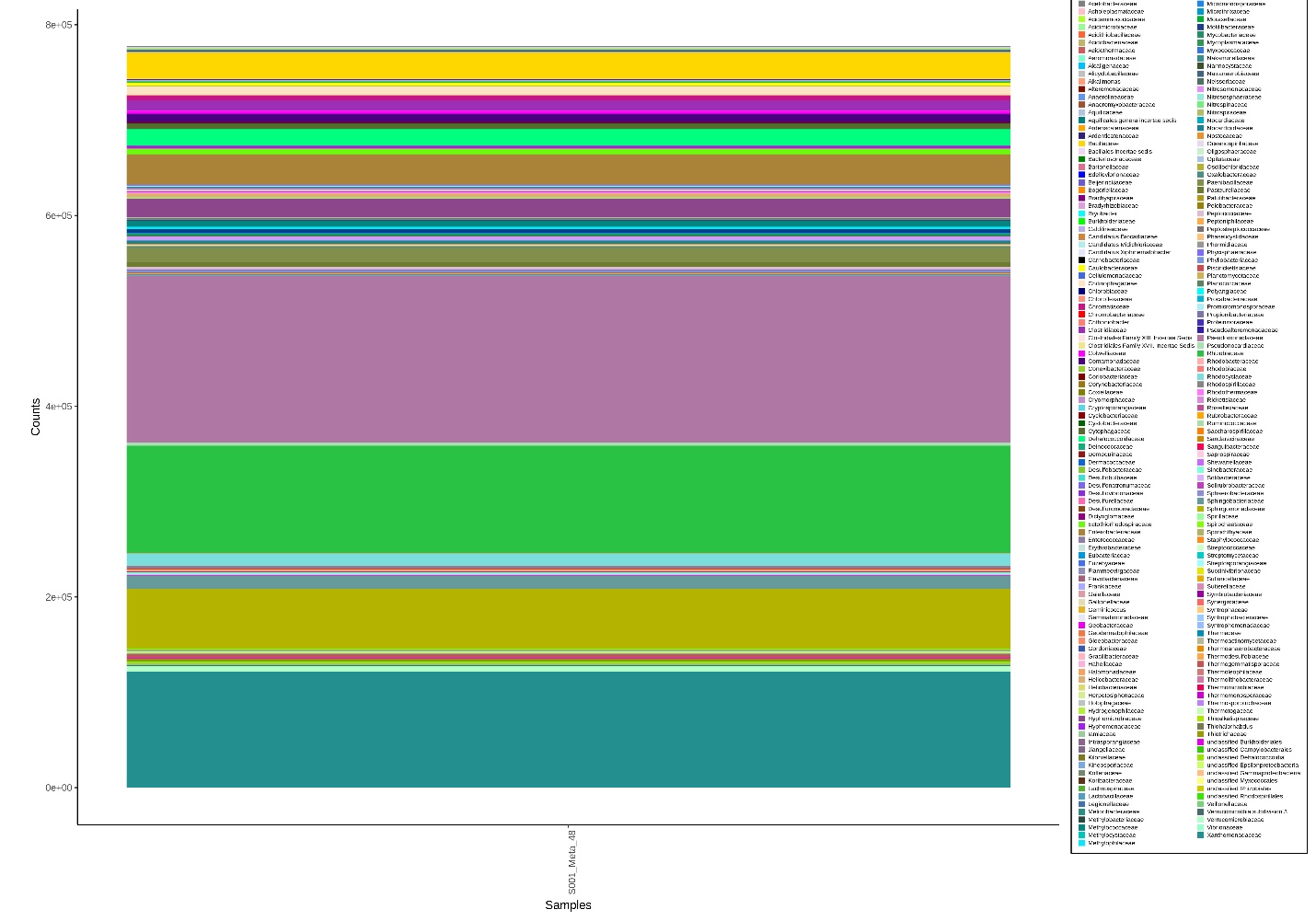


S1 Representation of soil indigenous microbial community on the family level for samples incubated with antibiotic at 30^o^C incubation (sample 48).


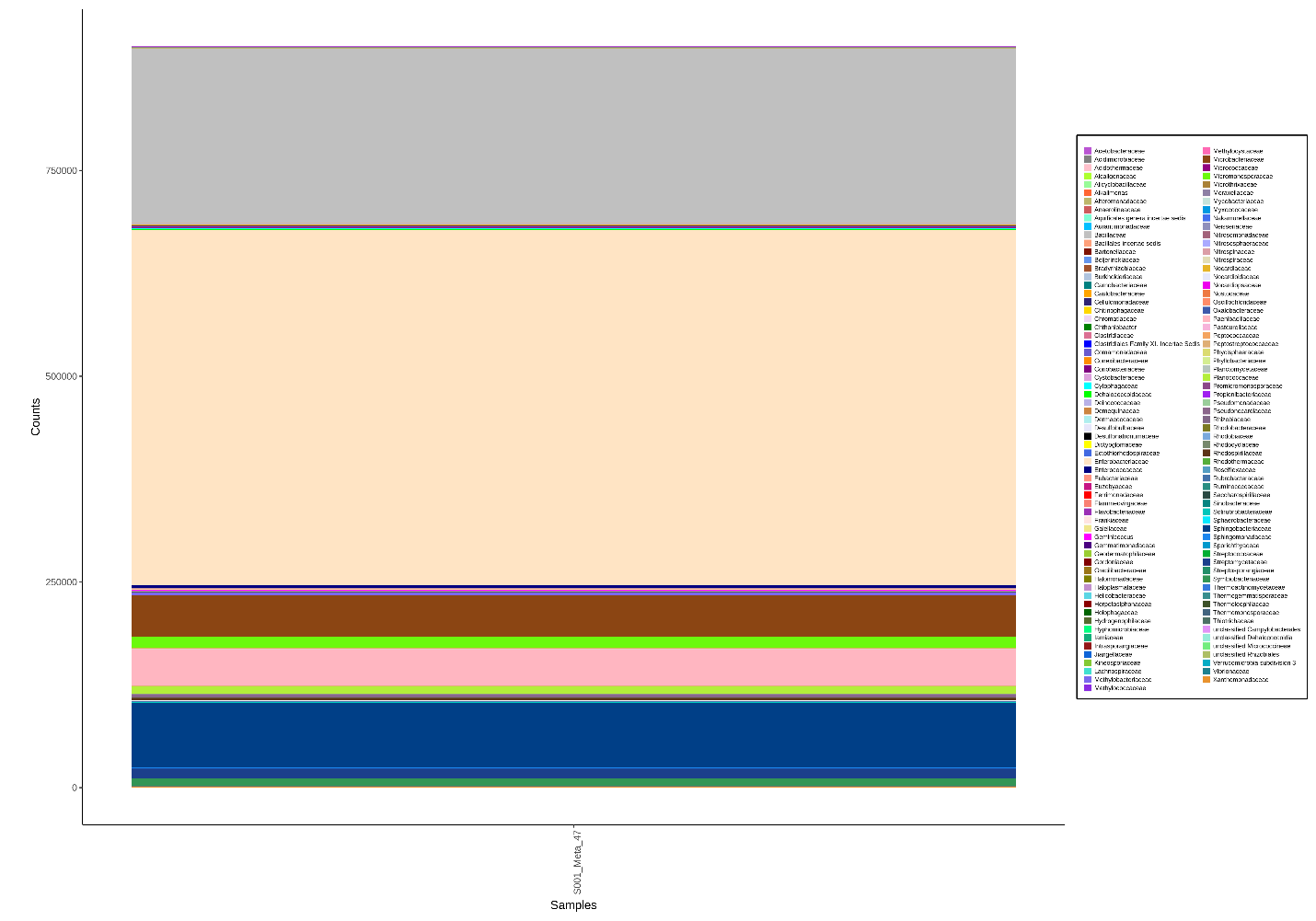


S2 Representation of soil indigenous microbial community on the family level for samples incubated with antibiotic at 40^o^C incubation (sample 47).


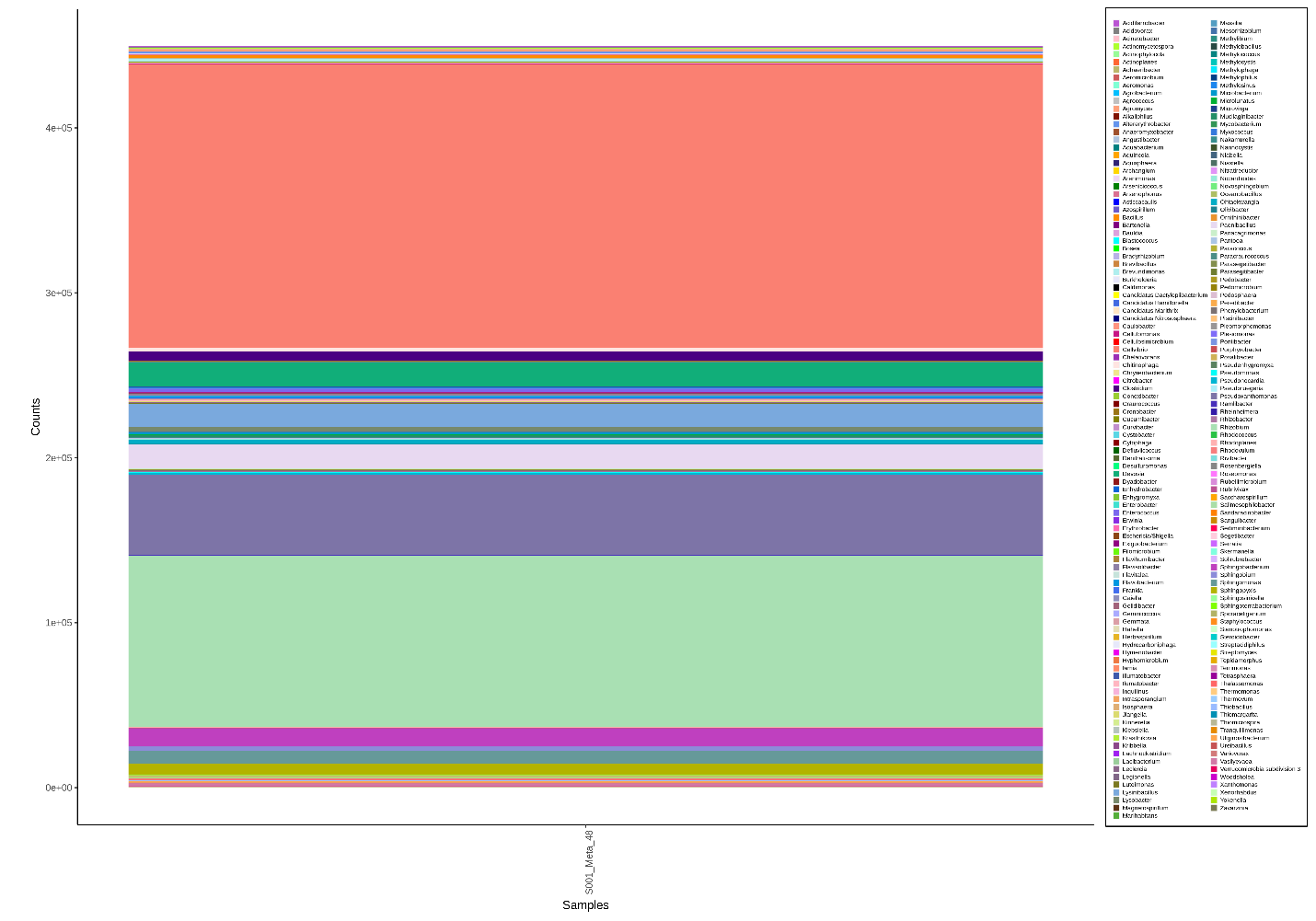


S3 Representation of soil indigenous microbial community on the genus level for samples incubated with antibiotic at 30^o^C incubation (sample 48).


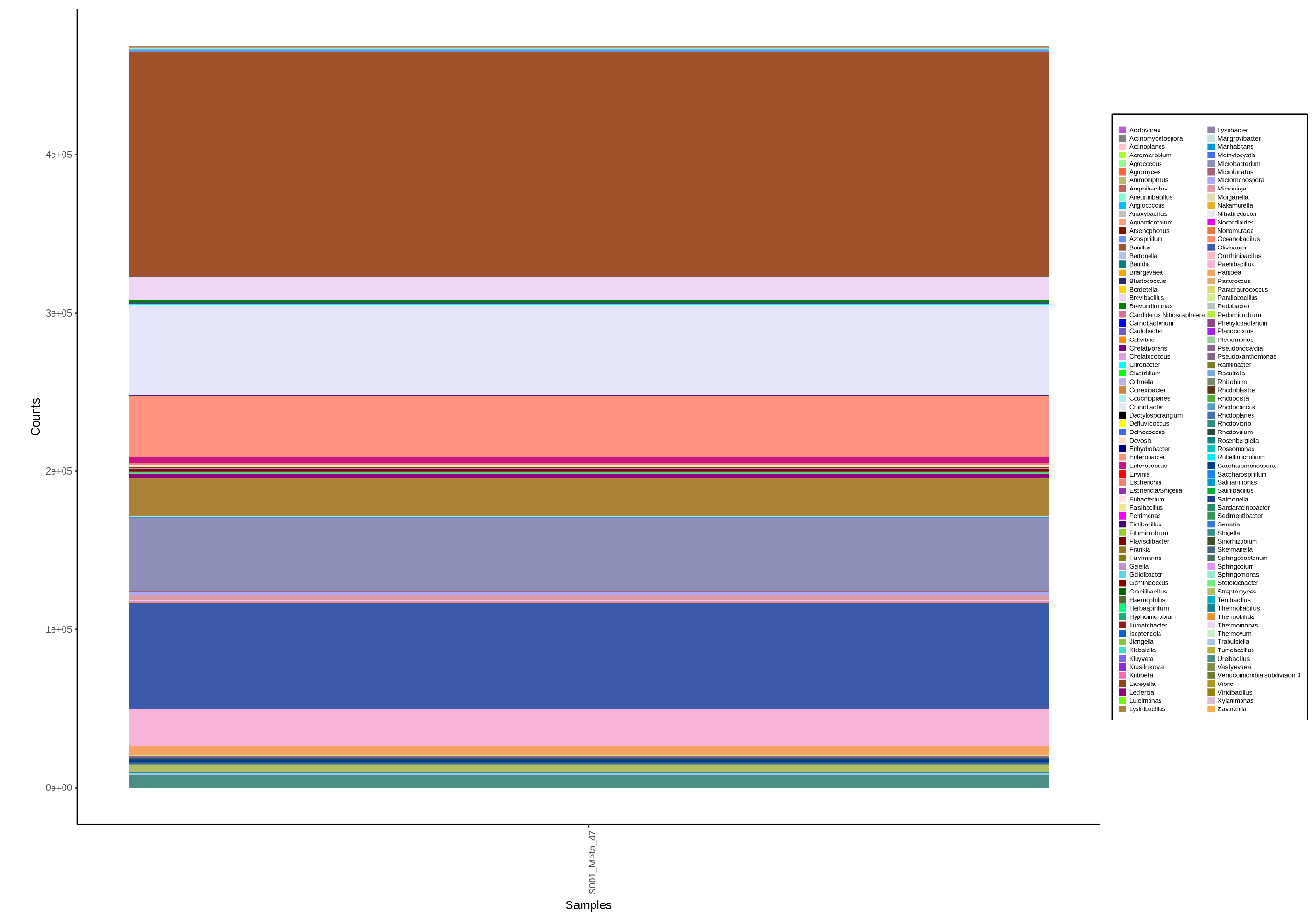


S4 Representation of soil indigenous microbial community on the genus level for samples incubated with antibiotic at 40^o^C incubation (sample 47).
